# Field evaluation of the potential for avian exposure to clothianidin following the planting of clothianidin-treated corn seed

**DOI:** 10.7717/peerj.5880

**Published:** 2018-11-07

**Authors:** Sean McGee, Melissa Whitfield-Aslund, Daiana Duca, Nicole Kopysh, Tereza Dan, Loren Knopper, Larry Brewer

**Affiliations:** 1 Bayer CropScience, LP, Research Triangle Park, NC, USA; 2 Stantec Consulting Ltd, Stoney Creek, ON, Canada; 3 Compliance Services International, Sisters, OR, USA

**Keywords:** Neonicotinoid, Birds, Treated seed, Clothianidin, Exposure

## Abstract

The objective of this study was to quantify consumption of clothianidin-treated corn seed by birds following standard planting practices. Based on post-planting seed counts on 21 fields in southwestern Ontario, Canada, between 29 and 813 seeds/ha (mean of 224 ± 167 (SD)) were estimated to remain on the soil surface immediately post planting (i.e., less than one seed per 10 m^2^). This represents between 0.03 and 1.2% of the total sown seeds. The number of seeds missing on each field on the third day after planting as a result of any process (e.g., removal by foraging birds or mammals or burial as a result of heavy rains) ranged from 0 to 136 seeds/ha (0 to 0.0136 seeds/m^2^). Behavior monitoring of individual birds and 24 h remote video surveillance were deployed to investigate how much of the treated seed remaining on the soil surface was consumed by birds. Spotting scopes were used to monitor the full duration of the field visits of 596 individual birds during morning hours for three consecutive days after planting on the 21 fields. Two birds were observed consuming treated seeds (one seed each) and three birds consumed seeds for which the treatment status could not be visually confirmed. Additionally, constant (24 h) video surveillance for 2–4 days immediately after planting was deployed at 24 areas where multiple treated seeds were found on the soil surface. Across 1,380 h of collected video footage (including both day and night periods), no birds were observed to consume any treated seeds. This study provides field evidence of two factors that determine exposure of birds to clothianidin-treated corn seeds: (1) standard sowing practices in Ontario are effective at burying treated seeds such that the proportion of sown seeds that remain on the soil surface after planting is low, and (2) birds monitored on these fields consumed very few of the clothianidin-treated corn seeds remaining on the soil surface after planting. As these results are dependent on planting techniques and seed characteristics, they are not necessarily applicable to other types of clothianidin treated seed.

## Introduction

Clothianidin and other systemic neonicotinoid insecticides such as imidacloprid, thiamethoxam, and dinotefuran are readily taken up by and circulated within plants, where they provide the plant with a period of systemic protection against biting and sucking pests (e.g., aphids, whiteflies, thrips, leafhoppers, scales, and leaf miners; Bonmatin et al., 2015; Environmental Protection Agency (EPA), 2017). This attribute allows clothianidin to be applied to seeds prior to planting and provide protection pre and post emergence for crops including canola, corn, potato, wheat, and several vegetable crops. Adoption of clothianidin as a corn seed treatment has been particularly strong in North America; a region where 1,846,680 and 88,715,700 ha of planted corn were reported in the most recent available report for Canada (2016) and the US (2017), respectively (Statistics Canada, 2016; United States Department of Agriculture National Agricultural Statistics Service (USDA-NASS), 2018).

Because treated seeds represent a potential food source for granivorous birds that forage on agricultural fields, concerns have been raised regarding the potential for avian consumption of treated seed and toxicological implications (Bro et al., 2016; Lopez-Antia et al., 2015; Millot et al., 2017; Turaga et al., 2016). In addition, recent publications have reported detectable residues of some neonicotinoids in plasma or liver samples obtained from wild birds (Hao et al., 2018; MacDonald et al., 2018). However, no studies have investigated the potential exposure and toxicological implications associated with the use of clothianidin as a corn seed treatment specifically, a major use of clothianidin in North America.

Bird behaviors and agronomic practices may limit exposure of birds to seed applied chemicals via consumption of treated seeds, particularly clothianidin-treated corn, under realistic field conditions (EPA, 2017). For seeds that have husks (e.g., millet, rice, sunflower, and sorghum), seed handling behavior (e.g., removing and discarding the seed husks to access the seed kernel) may reduce exposure to pesticides applied to the seed (Avery, Fischer & Primus, 1997). However, some seeds with husks are known, at least at times, to be eaten whole by certain bird species and even when a seed is husked the bird may still be exposed to pesticide residues while handling the seed. Corn seeds do not have a hull or husk; however, with an approximate mass of 225 mg, they are too big for small (e.g., 20 g) to medium (e.g., 100 g) sized passerine birds to consume whole (EPA, 2017; Benkman & Pulliam, 1988; Diaz, 1990, 1994), which is likely to limit their consumption by these birds. For birds that may still consume treated corn seed, standard agronomic practices such as coloring the seed and planting at depth may reduce exposure. Commercially treated seeds, such as clothianidin treated corn, are colored with a dye which previous studies have found may reduce the attractiveness of treated seeds that go undetected by a grower and remain on the surface after planting (Pank, 1976; Greig-Smith, 1987). Further, corn seeds are planted at depth (typically 38–51 mm below the soil surface to maximize root development) and therefore only seeds not appropriately planted (drilled) by equipment or spilled during loading of equipment and not cleaned up are expected to be available on the surface for consumption by birds. In their interim guidance on how to refine risk assessments for pesticide treated seeds, the Environmental Protection Agency (2016) suggested that an incorporation rate of 99% should be assumed for seeds (like corn) that are planted underground with an in-furrow planter or seed drill (i.e., only 1% of seeds should be considered available for bird consumption). Additionally, the product label for clothianidin-treated corn-seeds (e.g., Poncho® 600 [label]; Bayer CropScience, Research Triangle Park, NC, USA) mandates that spilled or exposed seeds must be incorporated into the soil or otherwise cleaned up from the soil surface.

While the agronomic and avian behavior factors described above may reduce exposure of birds to clothianidin residues on corn seeds, no prior field investigations have been published on the prevalence or toxicological impacts of bird consumption of clothianidin treated corn seed. To investigate how planting practices and bird behavior may affect potential exposure of birds to clothianidin-treated corn seeds under label compliant agronomic practices, this study was designed to (1) evaluate how many clothianidin-treated corn seeds remain available on the soil surface of monitored field plots after planting, and (2) observe how frequently these remaining seeds were consumed by birds. A total of 21 corn fields in southwestern Ontario, Canada, were observed in the first 3 days after planting to determine seed sowing efficiency, frequency, and duration of wild bird visits, and foraging behavior and food consumption on the fields by wild birds. Where possible, study plot conditions were selected to maximize seed availability and attractiveness to birds and thereby capture realistic upper-limits of exposure. For example, monitoring was carried out in the first 3 days after planting, when clothianidin residues are likely to be highest (clothianidin is water soluble and residues on seeds are expected to decline rapidly due to weathering) and seeds remain available (i.e., prior to germination). Additionally, avian monitoring focused on areas where seed availability was highest (e.g., turn rows and edge rows) and in field plots adjacent to suitable avian habitat (i.e., more likely to attract birds).

## Methods

This field study was conducted in accordance with US EPA Good Laboratory Practice Standards set forth under the Federal Insecticide, Fungicide, and Rodenticide Act (40 CFR, Part 160).

### Site selection

Potential field cooperators from Southern Ontario were identified from a list of growers that had purchased clothianidin-treated corn seed (Poncho®; Bayer CropScience, Research Triangle Park, NC, USA) at the highest available treatment rate (nominal 62.5 mg clothianidin/50 seeds, equivalent to nominal 1.25 mg clothianidin/seed). From this list, cooperator fields that were to be planted with clothianidin-treated seed were surveyed and selected for inclusion in the study based on a minimum distance of 800 m separating fields as well as agreement from growers that researchers could access the fields and standard agronomic practices would be followed without deviation. To maximize the potential for bird observation, only fields adjacent to suitable avian habitat (i.e., non-crop, non-pasture) were selected (i.e., where birds are more likely to reside when not actively foraging). The final selected sites included 10 fields in the vicinity of Guelph, Ontario and 11 fields in the vicinity of London, Ontario (see Fig. S1).

### Analytical verification of clothianidin concentration on seeds

A single commercial seed company delivered clothianidin-treated corn seeds directly to the cooperating growers and the seed treatment rate, lot number, and quantity delivered to each cooperating grower was verified. Samples of clothianidin treated corn seeds were collected by study team members from five different fields from seed bags just before planting, or from the seed hopper during planting, and shipped to Smithers Viscient in Wareham, MA for verification of the amount of test substance per seed. A subsample of 50 seeds from each of the sampled fields was analyzed for clothianidin by high-performance liquid chromatography (HPLC) with ultraviolet detection. The test substance was extracted from the seeds using acetonitrile-water (1:1 vol/vol) as the extraction solvent. HPLC was performed at 40 °C using a mobile phase of (A) 0.1% acetic acid in water and (B) 100% acetonitrile. The flow rate was 1.5 mL/min, with an injection volume of 5 μL and the eluent was monitored at 294 nm. The method was verified using an analytical standard (clothianidin) of 99.9% purity diluted in acetonitrile-water (1:1 vol/vol). Quality control (QC) samples spiked with known concentrations of clothianidin covering a specified range (low, mid, and high) were used to determine the accuracy of the method. The acceptable recovery limit for QC samples was set at 80–120%. The limit of quantification was 0.14 mg/mL.

### Planting of treated seeds

Planting was conducted in May to June by each grower using their personal planting equipment at planting rates that are typical for the region (i.e., 69,200–88,900 corn seeds/ha; Ontario Ministry of Agriculture, Food and Rural Affairs, 2017). Following typical “plant-to-moisture” practices for seed depth, planting depths were between 2.5 and 5 cm and varied based on the time since last rainfall and moisture retention of the soil. Growers were instructed not to alter their planting practices in any way. Rather, planting practices were left up to the individual growers without interference to ensure a realistic corn-planting scenario.

### Post-planting counts of seeds on the soil surface

The commercially available clothianidin-treated corn seeds evaluated in this study were purple due to treatment with an approved purple dye. Therefore, they were visually distinguishable from untreated (yellow) seeds. Immediately following planting, transects were established to count the number of corn seeds remaining on the soil surface, either left by the planter or because of an accidental spill event. Corn furrows were numbered and marked starting from one edge of each field. A total of 30–60 rows were selected to establish transects for counting seeds remaining on the soil surface (Table S1). Based on the seed row length, row spacing, and total number of rows included in each transect, the size of the transects that were directly monitored for corn seed counts ranged from 0.53 to 0.85 ha (Table S1). Given that the transects traversed across field centers, near-edge areas, and turn rows, the seed counts (per hectare) observed in these transects can be considered representative of the entire field. Based on the transect area, a density of seeds remaining on the soil surface was calculated for each field. Higher densities of seeds remaining on the soil surface are expected in turn and edge rows as compared to field centers. Transects were established to include edge areas and turn rows to ensure expected worst case areas were included in calculations. However, transects are not limited to edge areas and turn rows and also include field areas where fewer unincorporated seeds are expected. Therefore, the calculated values, reported as mean density, represent an average for the field and may not accurately reflect the highest unincorporated seed densities.

To perform the seed count, observers walked just to the side of the marked seed furrows and counted and recorded treated corn seeds visible on the soil surface in a swath that extended half the distance of the row spacing on both sides of the furrow. Mechanical tally recorders were used to ensure the count was not lost during the process. During the seed counts carried out immediately after planting (i.e., Day 0), observers marked seed spills or areas of multiple seeds remaining on the soil surface with small plastic plant tags, including a tag number and the number of seeds visible for a given spill. After 3 days, the corn seeds at each tagged location were recounted to determine the number of seeds that were missing.

### Characterization of avian species visiting study fields and observations of avian behavior on treated fields

One completely observable subplot that could be effectively monitored by an individual observer was established on each field and marked with flags prior to planting. These subplots were limited in size to ≤300 × 300 m at each field. The size and shape of each subplot differed slightly due to differences in topography in each field. However, in all cases, the subplot location in a field was chosen to represent an area where birds are most likely to forage based on the best adjacent avian habitat, highest potential for available seed by including turn-row area and adjacent field edge, and optimal blind location for observers to document behaviors without disrupting the birds.

For the first three consecutive days after planting, avian observations were carried out at each subplot by two observers who were experienced in identifying bird species in the region and familiar with normal avian behaviors and activities. These observations were carried out during morning hours that were considered to represent a period of high foraging activity (i.e., between 06:30 and 08:30 a.m.). One observer carried out avian census surveys, which consisted of monitoring the observable subplot with binoculars and identifying and recording the species and number of birds landing within the subplot. The other observer used a spotting scope to monitor a subset of individual birds on the field (one bird at a time) until they departed and recorded the time spent on the field, the number of corn seeds consumed and the treatment status of any consumed corn (i.e., treated or waste corn from previous year). Since only birds of a certain minimum size are likely to consume corn seeds (Benkman & Pulliam, 1988; Diaz, 1990, 1994), these behavioral observations focused on birds of sufficient size to consume corn seeds (e.g., red-winged blackbirds, grackles, blue jays, cardinals, crows, pheasants). Observers were instructed to record any mortality or impaired behavior if it occurred.

### Video monitoring observations

In addition to the observations carried out using binoculars and spotting scopes during morning foraging hours described above, remote, time-lapse, video equipment was deployed to provide constant (24 h) surveillance of select areas where multiple treated seeds were observed on the soil surface during the post-planting seed count (i.e., where birds are most likely to encounter visible treated corn seeds). One to three of these areas were selected for video monitoring at seven of the 11 London area fields and five of the 10 Guelph area fields, resulting in a total of 24 video monitored locations at 12 fields, with between two and 36 seeds monitored at each location. At each of the video-monitored locations, well camouflaged cameras were set up and focused on the identified areas of treated seeds, which were counted prior to initiating the recording. The camera was then left to record until the next day (between 20 and 24 h later), at which point the camera was stopped and the seeds were recounted. At most of the video-monitored locations, this process was repeated for three consecutive days and nights, within the first 3 days after planting (see Table S2). However, there were nine video-monitored locations that were monitored for shorter periods (1 or 2 days) due to heavy rainfall that interfered with camera operability (see Table S2). Likewise, at six locations, some monitoring occurred up to 4 days after planting due to weather conditions (see Table S2). If one or more seeds were missing at the end of the video recording session, then the videos were reviewed in detail to determine if any birds or animals interacted with the visible treated seeds over the duration of the surveillance period.

### Carcass searches

All study sites were searched and cleared of existing animal carcasses just prior to, during, or immediately after planting. For the first three consecutive days after planting, post-planting carcass searches were conducted after completion of the daily on-site bird observations. These searches were conducted along the study plot boundaries and just outside the boundaries along the field borders. Carcass searches consisted of four investigators searching established transects by foot in the defined areas, which included the turn-row area of the fields. The outside boundaries were searched with four individuals lined up approximately four m apart with one person at the field edge and the remainder spaced out in the adjacent habitat. Moving counter-clockwise around the field, each person searched a four m band of ground to their right, covering approximately 16 m of edge habitat in one trip around the field. Investigators then lined up with one person at the field edge and the other three spaced four m apart toward the field center. Again, moving counter-clockwise, each person searched a four m band to their left around the field perimeter. This was repeated with the search line moving 16 m toward the field center for each additional pass around the field until the entire field was searched.

## Results and Discussion

### Analytical verification of clothianidin concentration on seeds

The nominal clothianidin application rate for the commercially available seeds under consideration in this study (i.e., Poncho® 1250; Bayer CropScience, Research Triangle Park, NC, USA) was 1.25 mg active ingredient (a.i.) per seed. The measured loading per seed from a representative sample of seeds used on the study fields ranged from 1.2 to 1.4 mg a.i. per seed (i.e., 94–111% of the nominal rate). The recovery in four QC samples was within 99.5–104% of the fortified concentration demonstrating proper performance of the analytical method.

### Post-planting counts of seeds on the soil surface

The count-based estimate of number of treated seeds that remain on each field ranged from 29 to 813 seeds/ha, with a mean of 224 seeds/ha (Table 1). This corresponds to mean densities across the entire field—not considering the potential for higher densities in particular field regions (i.e., edge or turn rows)—of 0.0029–0.0813 seeds/m^2^ for individual fields and 0.0224 seeds/m^2^ as an average of all fields. This represents approximately 0.03–1.2% of the total sown seeds, based on standard planting rates of 69,200–88,900 corn seeds/ha (Ontario Ministry of Agriculture, Food and Rural Affairs, 2017). The variability between fields may relate to differences in planting equipment or soil characteristics (e.g., more seeds are likely to remain on the soil surface in heavy soil rather than light soil).

**Table 1:** Results of post-planting counts of seeds on the soil surface.

Field^a^	Seed count transect area (ha)	Seeds remaining on soil surface immediately after planting		Seeds missing from soil surface 3 days after planting	
		Total	Seeds/ha	Total	Seeds/ha
L1	0.53	19	35.8	3	5.66
L2*	0.73	146	200	32	43.8
L4	0.69	20	29.0	3	4.35
L5	0.69	159	230	7	10.1
L6*	0.69	154	223	ND^b^	ND^b^
L7	0.57	68	119	14	24.6
L10	0.85	241	284	17	20
L11*	0.69	133	193	37	53.6
L12*	0.65	185	285	40	61.5
L13	0.65	37	56.9	0	0
L14*	0.69	217	315	94	136
G1*	0.57	169	297	39	68.4
G2	0.69	57	82.6	5	7.25
G3*	0.69	208	301	61	88.4
G4*	0.69	561	813	33	47.8
G5	0.57	174	305	0	0
G7*	0.69	206	299	52	75.4
G10	0.57	69	121	3	5.26
G11	0.57	61	107	15	26.3
G12	0.57	69	121	0	0
G13	0.69	196	284	10	14.5
Mean ± SD	0.65 ± 0.08	150 ± 117	224 ± 167	23.3 ± 25.1	34.7 ± 36.8

**Notes:**

* Denotes sites that experienced heavy rainfall within the 3-day post-planting period.

a L prefix, London area sites; G prefix, Guelph area sites.

b ND, no data. A severe thunderstorm at this location washed out much of the drilled corn seed from the furrows; therefore, the grower re-disked the field, which buried all seed on the study site.

The highest observed mean density of unincorporated seeds for an entire individual field (0.0813 seeds/m^2^) is equivalent to the observation of less than one seed per 10 m^2^, while the lowest observed mean density of seeds for an entire individual field is equivalent to the observation of less than one seed per 300 m^2^. These densities may be insufficient to attract certain birds, as areas with high seed density are more attractive for birds than areas with only irregularly scattered seeds (De Leeuw et al., 1995). However, the reported densities are means of the post planting count transects and may not be fully representative of areas of the field (i.e., turn and edge rows) that generally have higher densities of unincorporated seeds. Further, the presence of other potential food sources on a treated field (e.g., waste corn from the previous year, weed seeds, and/or invertebrates) may attract birds to a treated field where they may subsequently encounter treated seeds. Therefore, additional monitoring was carried out in this study to determine how frequently treated corn seeds were actually consumed by birds.

The vast majority of seeds found on the soil surface after planting were found in the turn rows, and to a lesser extent along the first furrow down the edge of the field. The latter occurs because the outside planter wheel often rides on the untilled edge, which is rough and causes the planter to bounce more often than it does in the open field. Unincorporated corn was rarely found in the open field. Similarly, other field studies report a higher number of surface seeds on the headland of the field than at the center (Davis, 1974; Westlake et al., 1980; De Leeuw et al., 1995). In this study, each seed count transect contained turn rows at one end and a field edge along one side. Additionally, each seed count transect was limited in size to less than or equal to 0.85 ha (Table 1). Because the ratio of turn-rows and edge rows to open field will decrease as the field size increases, the mean number of treated seeds present on the soil surface after planting per hectare would likely be lower than observed in this study on larger field sizes (and vice versa).

Overall, these findings support the EPA (2016) recommendation that risk assessments for pesticide treated seeds could be refined by assuming that only 1% of seeds that are planted underground with an in furrow or drill seed planter (like corn) remain available on the surface for bird consumption. Based on the data available from the 21 fields monitored in this study, the 99% incorporation rate recommended by EPA (2016) is likely to provide a realistic upper limit of the number of treated corn seeds that are available on the soil surface for bird consumption.

The estimated number of seeds missing on each field, determined on the recount the third day after planting, ranged from 0 to 136 seeds/ha (0–0.0136 seeds/m^2^), with a mean of 34.6 seeds/ha (0.00346 seeds/m^2^) (Table 1). This excludes London field L6, as this field was re-disked due to heavy rain washing out much of the planted corn, which buried the treated seeds that were originally counted on the soil surface immediately post planting. This highest observed rate of missing seeds on the third day after planting (0.0136 seeds/m^2^) is equivalent to approximately one seed removed per 70 m^2^. These missing seeds may include seeds that were removed by birds or foraging mammals. However, as indicated in Table 1, the sites with the greatest number of missing seeds experienced heavy rainfall (i.e., where rainfall was observed to have moved and redeposited soil on a study plot). The number of missing seeds from sites experiencing heavy rain (i.e., 44–136 seeds/ha) was considerably higher than those without reported heavy rainfall (i.e., 0–26 seeds/ha) (Table 1). It is hypothesized that the greater number of seeds missing from the soil surface is due to the heavy rainfall burying seeds. This hypothesis is supported by observations of changes to the field topography from the heavy rain reported by study personnel and the low number of treated seeds seen consumed by wildlife in the video recording and visual observations. While rainfall may have contributed to a higher number of missing seeds it is not possible to distinguish the contribution of rainfall verse removal of seeds from birds and other wildlife that were not detected in the observations with full certainty. Sites that did not report heavy rainfall still had missing seeds that were not accounted for by observations of wildlife removal or typography changes that would suggest weather related removal.

### Characterization of avian species visiting study fields and observations of avian behavior on treated fields

Avian observations in the completely observable subplots on each of the 21 study fields were carried out from 06:30 to 08:30 a.m. each morning for the first three consecutive days after planting (i.e., 6 h × 21 fields = 126 h of avian observations in total). During this time, 1,882 occurrences of birds visiting one of the subplots were recorded as part of the overall avian census surveys. The birds most commonly reported to visit the fields were European starling (663/1882), common grackle (334/1882), and red-winged blackbird (160/1882). The complete census of bird species is presented in the Table S3.

Of the birds that were observed to visit one of the completely observable subplots, detailed behavioral observations (i.e., time spent on the field and number of corn seeds consumed) were recorded for a total of 596 birds, representing 25 species. The birds that were most commonly reported to visit the fields in the avian census surveys were also well represented in these detailed behavioral observations (i.e., European starling (181/596), common grackle (118/596), and red-winged blackbird (73/596)) (Table 2). A complete list of the birds that received detailed behavioral observation is provided in Table S3. The average duration spent on the treated fields by an individual observed bird was 3.3 min, resulting in a total of 1,987 min of detailed behavioral observations (Table 2).

**Table 2:** Summary of bird species monitored during the detailed behavioral observations.

Common name	Number of individual birds observed			Cumulative time that birds were observed on a field (min)	Average time spent per bird (min)
	Guelph fields	London fields^a^	Total		
European starling	105	76	181	487	2.7
Common grackle	97	21	118	287	2.4
Red-winged blackbird	22	51	73	160	2.2
American robin	20	19	39	113	2.9
American crow	36	0	36	201	5.6
Pigeon (rock dove)	30	0	30	238	7.9
Brown-headed cowbird	0	16	16	33.5	2.1
Horned lark	15	0	15	37	2.5
Other^b^	28	60	88	431	4.9
Total	353	243	596	1,987	3.3

**Notes:**

a There were two London sites on which field observations were not possible on Day 2 and Day 3 (site L6) and on Day 3 (site L14) due to heavy rainfall and severe thunderstorms.

b “Other” includes all species observed on a study plot fewer than 15 times during the observation period. For the London sites, this included a total of 21 species. For the Guelph sites, this included a total of eight species.

Of the 596 birds monitored during the behavioral observations, the majority of them (95%) were not observed consuming corn seeds while present on the observable subplots of each field. For those that did consume corn seeds, untreated waste corn left over from the previous season (much of which was still on the cob and had become soft due to over-wintering) was more frequently consumed than treated corn seeds (Table 3). Specifically, 21 birds, representing four different species, were observed picking through the waste seed until they found one that was soft enough to crush in their beaks, and then eating that seed or flying off with it (Table 3). Additionally, two birds (one blue jay (*Cyanocitta cristata*) and one American crow (*Corvus brachyrhynchos*)) were observed to consume clothianidin-treated corn seeds (Table 3). For both birds, this consumption was limited to a single treated seed (Table 3). Additionally, seeds with an unknown treatment status (i.e., for which treatment status could not be visually confirmed by the observer) were observed to be consumed by one Canada goose (*Branta canadensis*) (seven seeds), one blue jay (*C. cristata*) (one seed), and one red-winged blackbird (*Agelaius phoeniceus*) (one seed) (Table 3).

**Table 3:** Summary of birds observed to consume seeds on the London and Guelph study sites during detailed behavioral observations.

Field location	Species	Number of individual birds observed consuming corn	Number of corn seeds consumed		
			Treated	Unknown^b^	Untreated
London^a^	Red-winged blackbird	1	0	1	0
	Common grackle	1	0	0	5
	Brown-headed cowbird	1	0	0	1
Guelph	Blue jay	1	1	1	0
	Horned lark	1	0	0	2
	Canada goose	1	0	7	0
	Common grackle	9	0	0	>7^c^
	House sparrow	9	0	0	>1^d^
	American crow	1	1	0	0
Total		25	2	9	>15^e^

**Notes:**

a There were two London sites on which field observations were not possible on Day 2 and Day 3 (site L6) and on Day 3 (site L14) due to heavy rainfall and severe thunderstorms.

b Seed treatment status could not be visually confirmed.

c Four of the nine common grackle consumed an undetermined amount of non-treated seeds.

d The number of non-treated seeds consumed by the nine individual house sparrows could not be determined.

e The total number of non-treated corn seeds consumed could not be determined as a result of the two bird species which consumed an indeterminate amount.

The low number of observations of birds consuming treated corn seeds is consistent with the low density of treated seeds remaining on the soil surface (i.e., 0.0029–0.0813 seeds/m^2^). It is also possible that birds may have avoided treated seeds due to appearance, palatability, and/or in response to learned behavior from sublethal effects from prior exposure (De Leeuw et al., 1995; Avery, Decker & Fischer, 1994; Lopez-Antia, Ortiz-Santaliestra & Mateo, 2014); however, avoidance behaviors were not directly investigated in this study.

No abnormal behaviors or mortalities were detected for any birds while present on the observable subplots of each field. However, these observations were limited to the time that birds were present on the observable subplot. The likelihood of acute toxicity based on available acute avian toxicity data for clothianidin and the number of treated seeds that individual birds were observed to consume is presented in the ‘Risk evaluation’ section, below.

### Video monitoring observations

Continuous video monitoring was deployed at 24 locations across 12 fields (seven London area fields and five Guelph area fields) for 2–4 days immediately after planting (see Table S2). Each camera focused on an area where multiple seeds remained visible on the soil surface (between two and 36 seeds in frame). In total, 1,380 h of video footage (including both day and night periods) were collected. Based on seed counts conducted before and after video monitoring, it was possible to observe that there were a total of three occasions when a single seed was removed from a monitored area, and one occasion when a seed was cracked, but not consumed (see Table S2). A review of the collected video footage indicated that two of the three removed seeds were taken by mice. The third seed removal occurred during a heavy rain/wind storm, which caused the camera to malfunction. Therefore, it was not possible to identify whether the seed was displaced by the weather or by an animal. However, it was unlikely to have been removed by a bird because this removal occurred during the night, and granivorous birds do not generally feed at night. It was also not possible to determine whether an animal had handled the single observed cracked seed, because the camera was blown over by the wind during that observation period. Therefore, it is possible that a bird attempted to eat this seed but was ultimately deterred from consuming it. Overall, across 1,380 h of video monitoring (including both day and night periods) collected at 24 locations where multiple seeds were available over 2–4 day periods immediately after planting, no birds were observed eating treated seed.

### Carcass searches

No bird carcasses were located during the carcass searches conducted on each field at the end of each day of daily bird observations (i.e., each day during the 3-day post-planting observation period). The absence of bird carcasses during the 3-day search period post-planting does not necessarily mean that mortality did not occur. It is generally recognized that several factors can contribute to underestimation of bird mortalities based on recovered carcasses including: (1) birds may die outside of the treated field or the area; (2) carcasses may be removed from the search area by scavengers before they are detected (3) the efficiency of searchers at detecting carcasses and; (4) the duration over which monitoring is completed (Balcomb, 1986; Prosser, Nattrass & Prosser, 2008). However, the absence of carcass discoveries during the daily searches provides one line of evidence that is consistent with the low number of observations of treated seed consumption by birds observed in this study.

### Risk evaluation

For the small number of birds that did consume treated seeds or seeds with an unknown treatment status in this study, acute oral toxicity data for clothianidin can be used to illustrate the potential significance of the observed acute clothianidin exposure. This analysis only considers acute lethality as a result of exposure from consumption of treated seeds. The field investigation described herein does not address potential exposure via other sources or the potential for chronic exposure or effects. Sublethal impact (e.g., weight loss from reduced food consumption or ataxia) that may reduce the fitness of a bird are expected to occur at exposure below levels resulting in mortality. However, the use of LD50 values for assessing risk is a standard practice for regulatory authorities around the world including EPA and the Canadian PMRA, and is used here to characterize the risk of the worst outcome (death) from acute exposure.

Quantitative acute oral toxicity data are available for four species of birds (see Table S4), with the reported LD50s ranging from 414 to >2,000 mg a.i/ kg body weight. Based on the acute oral toxicity data for the most sensitive bird species tested (i.e., 414 mg a.i./kg bw, reported for the red-winged blackbird (*A. phoeniceus*)) the number of treated seeds that would constitute an acute median lethal dose for the birds observed consuming treated seeds or seeds with an unknown treatment status was calculated and compared to the number of seeds that they were observed to consume (Table 4). Based on the observed treated seed consumption, one blue jay and one American crow were exposed to approximately 3.5% and 0.6%, respectively, of the LD50 dose (Table 4). Additionally, if it is assumed that all the consumed seeds of unknown status were actually treated seeds, then the exposure for the blue jay would increase to 7% of the LD50, and one red-winged blackbird and one Canada goose would also have been exposed to 5.2% and 0.5% of the LD50, respectively. This study does not provide a full account of the dietary exposure to clothianidin of individual birds for an entire day (i.e., the daily dose), rather only a small portion of the foraging time of each individual bird was observed. However, given that most individual birds were not observed consuming treated seeds at all and that the treated seeds did not appear to be an attractive food item to birds (even in areas where seed density was highest/most attractive), it is expected that overall clothianidin exposure due to consumption of treated corn seeds will be minimal for most birds. Similarly, although this study design does not provide quantitative information regarding the likelihood of chronic exposure of birds to clothianidin treated corn seeds, the low occurrence of treated seed consumption in this study suggests that the potential for such chronic exposure via consumption of treated seeds in the field is also unlikely. Chronic exposures to treated seeds are also expected to be limited by the rapid germination of commercial seeds and the water solubility of clothianidin (327 mg/L, EPA, 2017), which can result in a rapid dissipation of clothianidin from the seed coating in the presence of water. This study does not provide data on the potential acute or chronic exposure to clothianidin via routes other than consumption of treated seeds.

**Table 4:** Risk evaluation of consumed corn seeds based on avian acute oral toxicity.

Species	Body weight (kg)^a^	Number of corn seeds consumed		Dose^b^ (mg a.i./kg bw)	Fraction of LD50 (%)^c^
		Treated	Unknown		
Red-winged blackbird	0.06	0	1	0–22	0–5.2
Blue jay	0.09	1	1	14–29	3.5–7.0
American crow	0.55	1	0	2.4	0.6
Canada goose	4.5	0	7	0–2	0–0.5

**Notes:**

a For the red-winged blackbird, the body weight is represented by the median value of the body weight range reported by Rosenthal (2004); for the blue jay and American crow, values were taken from Poole (1938) for the Canada goose value was taken from BC MOE (British Columbia Ministry of the Environment) (1996).

b = (Seed dose * Total number of seeds consumed)/Body weight^a^. The seed dose is the mean measured clothianidin concentration per seed (1.3 mg a.i./seed). If a range is shown, the lower limit represents only exposure via consumption of treated seeds and the upper limit represents estimated exposure if all seeds with unknown treatment status are assumed to be treated seeds.

c = Potential exposed dose^b^/The lowest available acute avian LD50 of 414 mg a.i./kg-bw for the red-winged blackbird. If a range is shown, the lower limit represents only exposure via consumption of treated seeds and the upper limit represents estimated exposure if all seeds with unknown treatment status are assumed to be treated seeds.

## Conclusion

The objectives of this study were (1) to evaluate how many clothianidin-treated corn seeds remain available on the soil surface after planting and (2) to observe how frequently these remaining seeds were consumed by birds under realistic label use conditions. Based on this study, the mean density of treated corn seeds remaining on the soil surface of individual fields immediately after planting was low (0.0029 to 0.0813 seeds/m^2^) and represented only a small proportion (in most cases, less than 1.0%) of the total sown seeds. Furthermore, the treated corn seed that remained available on the soil surface after planting was not an attractive food source to birds. As these results are dependent on planting techniques (e.g., seeds planted at depth) and seed characteristics (e.g., large seed size), they are not necessarily applicable to other types of clothianidin treated seed (e.g., cereal grains).

## Supplemental Information

10.7717/peerj.5880/supp-1 Supplemental Information 1 Map showing the location of London and Guelph, Ontario, Canada. ©2018 Google. Image NOAA. Image Landsat/Copernicus. Data SIO, NOAA, U.S. Navy, NGA, GEBCO.

10.7717/peerj.5880/supp-2 Supplemental Information 2 Characteristics of seed counting transects on each study field.

10.7717/peerj.5880/supp-3 Supplemental Information 3 Observations from video recordings.

10.7717/peerj.5880/supp-4 Supplemental Information 4 Summary of avian observations across all 21 completely observable subplots from 06:30 to 08:30 a.m. for the first three consecutive days after planting.

10.7717/peerj.5880/supp-5 Supplemental Information 5 Clothianidin avian acute oral toxicity.

10.7717/peerj.5880/supp-6 Supplemental Information 6 The measured amount of clothianidin per 50 seeds from the 5 samples collected in the field at 5 different study site locations.

10.7717/peerj.5880/supp-7 Supplemental Information 7 Poncho treated seed delivered by seed company representatives to cooperating growers.

10.7717/peerj.5880/supp-8 Supplemental Information 8 Planting dates, types of planters used on London and Guelph area study sites in 2004.

## Additional Information and Declarations

### Data Availability

The following information was supplied regarding data availability: The raw data are provided in the Supplemental Files.
