# Supplementary figures and images for: Field evaluation of the potential for avian exposure to clothianidin following the planting of clothianidin-treated corn seed
Source: PeerJ. 2018 Nov 7;6:e5880. doi: 10.7717/peerj.5880 (PMC6228585; doi:10.7717/peerj.5880)

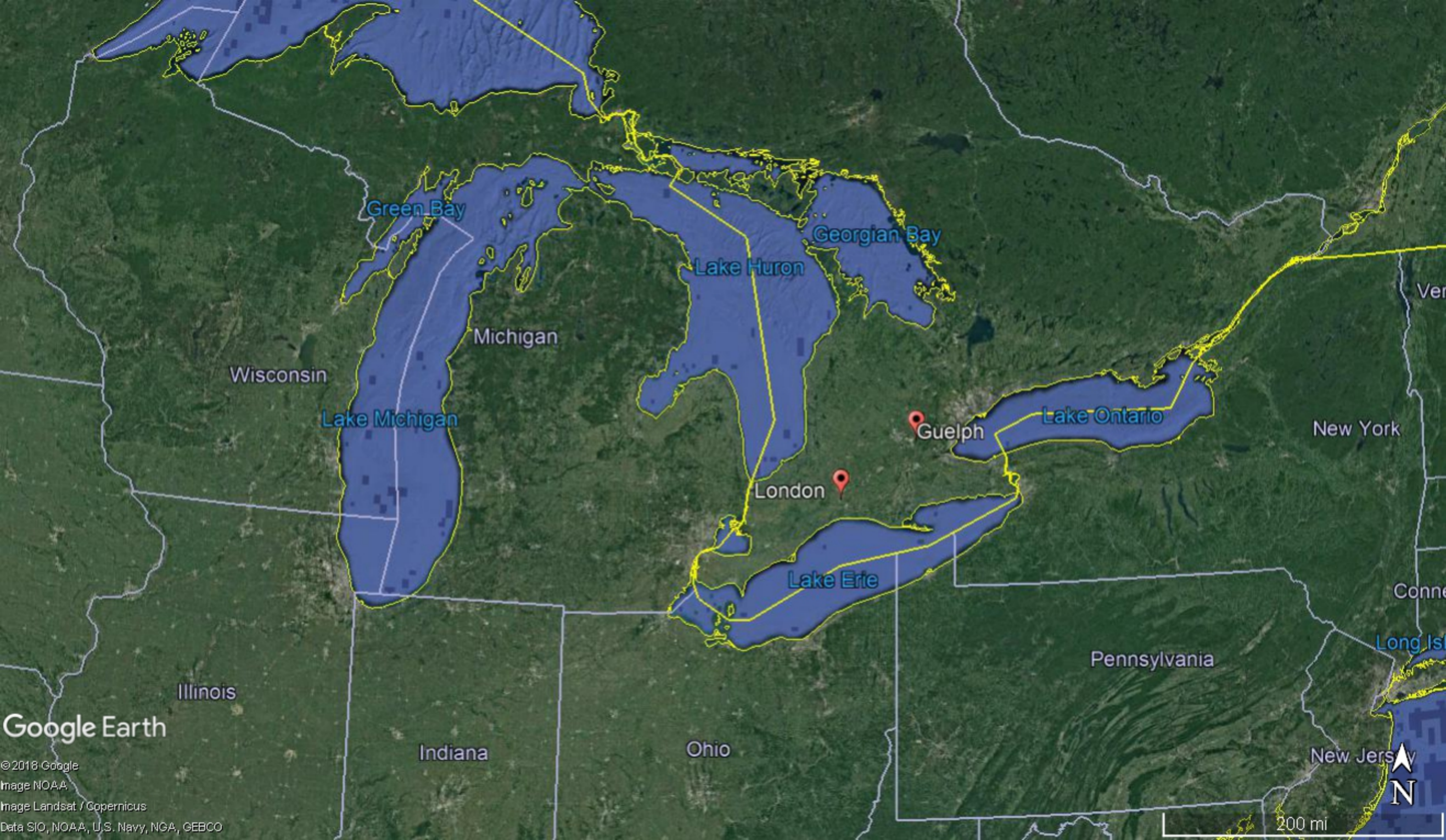

Google Earth

© 2018 Google  
Image NOAA  
Image Landsat / Copernicus  
Data SIO, NOAA, U.S. Navy, NGA, GEBCO

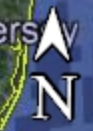

Supplement: Supplemental Information 1 — ©2018 Google. Image NOAA. Image Landsat/Copernicus. Data SIO, NOAA, U.S. Navy, NGA, GEBCO. [file peerj-06-5880-s001.pdf]
